# Wavelength-Adaptive Dehazing Using Histogram Merging-Based Classification for UAV Images

**DOI:** 10.3390/s150306633

**Published:** 2015-03-19

**Authors:** Inhye Yoon, Seokhwa Jeong, Jaeheon Jeong, Doochun Seo, Joonki Paik

**Affiliations:** 1 Department of Image, Chung-Ang University, 84 Heukseok-ro, Dongjak-gu, Seoul 156-756, Korea; E-Mails: inhyey@gmail.com (I.Y.); sukhwa88@gmail.com (S.J.); 2 Department of Satellite Data Cal/Val Team, Korea Aerospace Research Institute, 115 Gwahangbo, Yusung-Gu, Daejon 305-806, Korea; E-Mails: jjh583@kari.re.kr (J.J.); dcivil@kari.re.kr (D.S.)

**Keywords:** image dehazing, image defogging, image enhancement, unmanned aerial vehicle images, remote sensing images

## Abstract

Since incoming light to an unmanned aerial vehicle (UAV) platform can be scattered by haze and dust in the atmosphere, the acquired image loses the original color and brightness of the subject. Enhancement of hazy images is an important task in improving the visibility of various UAV images. This paper presents a spatially-adaptive dehazing algorithm that merges color histograms with consideration of the wavelength-dependent atmospheric turbidity. Based on the wavelength-adaptive hazy image acquisition model, the proposed dehazing algorithm consists of three steps: (i) image segmentation based on geometric classes; (ii) generation of the context-adaptive transmission map; and (iii) intensity transformation for enhancing a hazy UAV image. The major contribution of the research is a novel hazy UAV image degradation model by considering the wavelength of light sources. In addition, the proposed transmission map provides a theoretical basis to differentiate visually important regions from others based on the turbidity and merged classification results.

## 1. Introduction

Acquisition of high-quality images is an important issue in securing visual information of unmanned aerial vehicle (UAV) platforms. However, most UAV images are subject to atmospheric degradation. Among various factors of image degradation, haze or fog in the atmosphere results in color distortion, which can lead to erroneous analysis of important object regions. In reviewing the literature, dark channel prior-based defogging methods are first analyzed. We then address issues of the limitations and problems of the dark channel prior-based methods and justify the need of the wavelength-adaptive model of hazy image formulation.

A major approach to dehazing utilizes the dark channel prior that decomposes an image into the hazy and haze-free regions. The dark channel prior is a kind of statistics of the haze-free outdoor images. It is based on the assumption that most local patches in a haze-free outdoor image contain some pixels that have very low intensities in at least one color channel [1]. In order to solve the color distortion problem of He's method, Yoon *et al.* proposed an edge-based dark channel prior and corrected color distortion using gradient-based tone mapping [2]. Xie *et al.* used a combined bilateral and denoising filter to generate the transmission map [3]. Gao *et al.* further reduced halo effects using a guided filter and applied the maximum visibility to control the turbidity [4]. Park applied the weighted least squares-based edge-preserving smoothing filter to the dark channel prior and performed multi-scale tone manipulation [5]. Kil *et al.* combined the dark channel prior and local contrast enhancement to remove haze and correct color at the same time [6]. Yeh *et al.* proposed a fast dehazing algorithm by analyzing the haze density based on pixel-level dark and bright channel priors [7]. He *et al.* extended their original work by introducing the atmospheric point spread function to restore sharper dehazed images [8]. Shi estimated the amount of hazy components by detecting the sky region using the dark channel prior [9]. Although the proposed method shares a similar framework with Shi's method, it detects the sky region using various features instead of the dark channel prior. In addition, wavelength-adaptive enhancement is another contribution of the proposed work. Long *et al.* proposed an improved dehazing algorithm using the dark channel prior and a low-pass Gaussian filter for remote sensing images [10].

Although the dark channel prior has played an important role in various dehazing algorithms, the related methods suffer from color distortion and edge degradation, since they do not consider the wavelength characteristics in the image degradation model. In order to solve these problems, there were various dehazing algorithms without using the dark channel prior. Narasimhan *et al.* acquired two differently-exposed images for the same scene to estimate the amount of light penetration and the depth [11,12]. Shwartz *et al.* used two differently-polarized filters to reduce the polarized hazy component [13]. The use of two images makes these algorithms computationally expensive and impossible to be implemented in real-time. Schechner *et al.* proved that noise is amplified in a distant object region, because of the low transmission ratio [14]. Fattal measured the reflection ratio and performed dehazing under the assumption that directions of reflection should be identical at the same location [15]. Tan observed that the amount of haze or fog and contrast depend on the distance from the camera [16], and Kratz estimated statistically-independent components of image albedo and distance using Markov random fields [17]. Although three-dimensional (3D) geometry provides more reliable intensity of haze or fog, the related algorithms cannot be implemented in real-time video systems, because of the complex geometric transformation steps. Additional dehazing algorithms based on physical characteristics of haze or geometric information of the imaging process were proposed in [18, 19].

The third group of dehazing algorithms can be categorized as an application-specific approach. Gibson applied the dehazing algorithm before and after video compression to reduce coding artifacts [20], and Chiang utilized the characteristics of the underwater image to modify the dark channel prior and proposed a dehazing algorithm with color and wavelength correction [21]. Pei proposed a combined color correction and guided filtering to remove blue shift in the night image [22]. Wen computed the scattering and transmission factors of light in underwater images and successfully removed haze using the difference of light attenuation Yoon *et al.* proposed color preserved defogging algorithms by considering the wavelength dependency [23, 24].

The common challenge of existing dehazing methods includes color distortion and the associated high computational load to correct colors without considering the wavelength dependency. In order to solve this problem, we first present a wavelength-adaptive hazy UAV image degradation model, and a spatially-adaptive transmission map is generated using geometric classes and dynamic merging according to the model. The proposed wavelength-adaptive transmission removes hazy components without color distortion. As a result, the proposed algorithm needs neither additional optical equipment nor *a priori* distance estimation. The proposed dehazing method can significantly increase the visual quality of an atmospherically-degraded UAV image in the sense of preserving color distortion without the halo effect. Although the proposed model is established primarily for UAV images, it can be used to enhance any aerial hazy photography, video surveillance systems, driving systems and remote sensing systems.

This paper is organized as follows. Section 2 describes the wavelength-adaptive UAV image formation model, and Section 3 presents the proposed single image-based dehazing approach. Experimental results are given in Section 4, and Section 5 concludes the paper.

## 2. Wavelength-Adaptive UAV Image Formation Model

Rayleigh's law of atmospheric scattering provides the relationship between the scattering coefficient β and the wavelength λ, which is defined as [11]:

(1) $$\beta (\lambda )\propto \frac{1}{\lambda^{\gamma }}$$

where 0 ≤ γ ≤ 4 depends on the size of particles distributed in the atmosphere. In the haze-free atmosphere, haze particles can be considered to be sufficiently smaller than the wavelength of the light, and γ takes its maximum value, which is γ = 4. More specifically, if γ increases, the amount of scattering by haze particles becomes more dependent on the wavelength. On the other hand, in the hazy atmosphere, the size of haze particles is larger than the wavelength of the light, and γ takes its minimum value, such as γ ≈ 0. For a small γ, the amount of scattering becomes less dependent on the wavelength of light.

**Figure 1.**
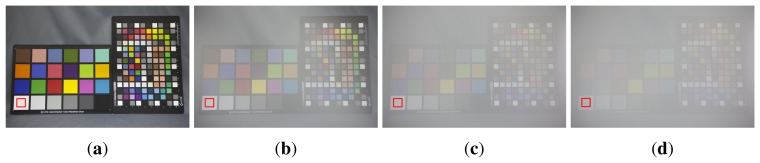
Test images acquired from the same indoor scene with a different amount of steam generated by a humidifier: (**a**) the haze-free image without steam; and (**b**–**d**) the hazy images with different amounts of steam.

If there is a sufficiently large amount of fog or haze, the amount of scattering light is assumed to be uniform regardless of the wavelength. This assumption is proved by the simple experiment based on the theory of Narasimhan [11,12]. In order to observe the wavelength-dependent scattering, Figure 1 shows four images acquired from the same indoor scene with different turbidities generated by the different amount of steam using a humidifier.

**Figure 2.**
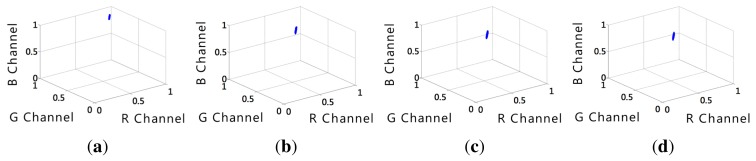
The distribution of the RGB color of the red rectangular regions in Figure 1.

In Figure 1, the white region enclosed by the red rectangles contain the pure white color, and color distributions of the red rectangular regions in the four images were used. Theoretically, pure white regions, as shown in Figure 1a, have unity in all RGB color channels. However, the real values are not exactly the same as unity, but are close to unity because of noise. The color distribution appears as a “point” in the three-dimensional RGB space, as shown in Figure 2a. On the other hand, as the amount of haze increases in the atmosphere, the distribution becomes elongated ellipsoids, as shown in Figure 2b–d, since the turbidity of haze decreases the brightness of the object by scattering.

**Figure 3.**
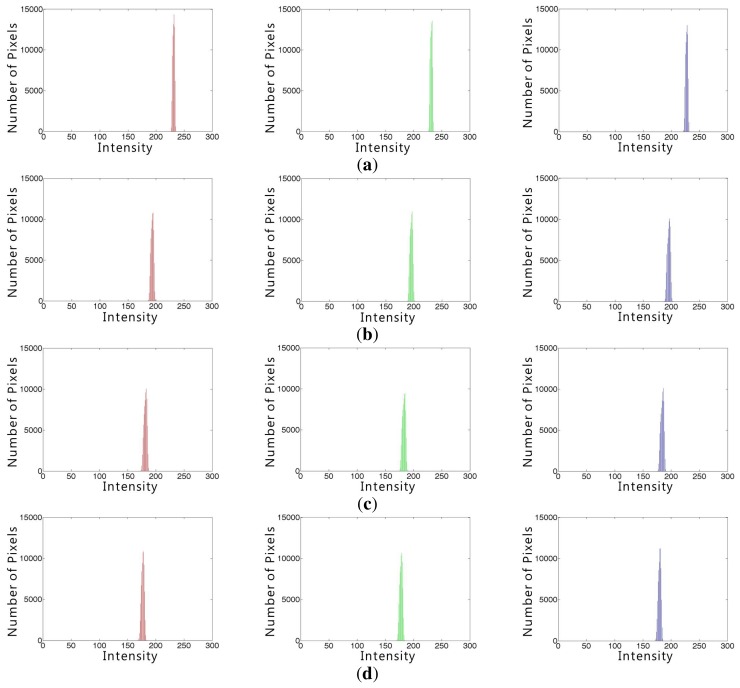
The RGB color histograms of the white regions in Figure 1: (**a**) red, green and blue (from left to right) color histograms of the white regions of the haze-free image shown in Figure 1a; and (**b**–**d**) red, green and blue color histograms of the hazy images shown in Figure 1b–d.

More specifically, Figure 3 shows RGB color histograms in four white regions enclosed by the red rectangle in Figure 1 and supports the observation in Figure 2.

This simple experiment shows that the amount of scattering depends on the turbidity of haze, which follows the observation by Narasimhan [12]. For this reason, restoration of the original color and brightness of an object is a challenging problem in the hazy environment.

Since the turbidity of a hazy image varies by the size of atmospheric particles and the distance of an object, the dehazing process should be performed in a spatially-adaptive manner. Let a small region with homogeneous color and brightness, or simply a cluster, have mean brightness values in RGB color channels as *C_r_, C_g_* and *C_b_*. The effect of scattering can be estimated from the quantity of *C_r_, C_g_* and *C_b_* using the color alignment measure (CAM) proposed in [25]:

(2) $$L=\lambda_{r}\lambda_{g}\lambda_{b}/\sigma_{r}^{2}\sigma_{g}^{2}\sigma_{b}^{2}$$

where λ*_r_*, λ*_g_* and λ*_b_* represent eigenvalues of the RGB color covariance matrix of the white region and $\sigma_{r}^{2}$ , $\sigma_{g}^{2}$ and $\sigma_{b}^{2}$ the corresponding diagonal elements of the covariance matrix. *L* represents the amount of correlation among RGB components.

The CAM represents the degree of dispersion among RGB channels and increases if the dispersion becomes more dependent on the wavelength. Table 1 summarizes mean brightness values *C_r_, C_g_* and *C_b_* of RGB components and CAM values of four white regions in Figure 1. As shown in the table, the more turbid the atmosphere, the less the scattering is affected by the wavelength.

A white region is suitable to estimate the CAM parameter, since it contains pan-chromatic light. Table 1 summarizes RGB components of mean brightness values, *C_r_, C_g_* and *C_b_* and the corresponding CAM values of four white regions in Figure 1. As shown in the table, as the turbidity increases, the scattering amount becomes independent of the wavelength.

**Figure 4.**
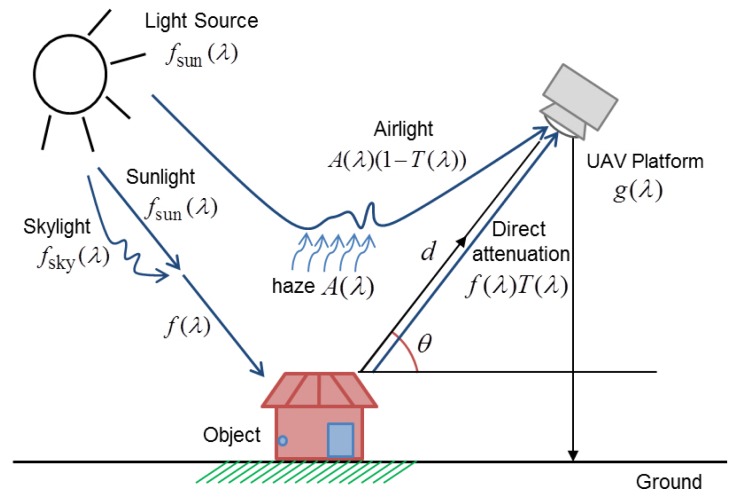
The proposed wavelength-adaptive UAV image formation model in a hazy atmosphere acquired by a UAV platform. *f*_sun_(λ) and *f*_sky_(λ) respectively represent the sun and sky light. The sky light *f*_sky_(λ) represents the light component that is scattered in the atmosphere.

Based on this experimental work, the proposed degradation model of wavelength-dependent hazy unmanned aerial vehicle (UAV) images can be considered as an extended version of Yoon's work in [24], as shown in Figure 4.

The mathematical expression of the proposed UAV image formation model is given as:

(3) g(λ)=f(λ)T(λ)+A(λ)(1−T(λ))

where *g*(λ) represents the hazy image of wavelength λ ∈ {λ_red_, λ_green_, λ_blue_}, *f*(λ) = *f*_sun_(λ) + *f*_sky_(λ) is the original haze-free image, *A*(λ) is the atmospheric light and *T*(λ) is the transmission map. As described in Equation (3), *f*(λ) is the combination of the scattering light in the atmosphere *f*_sun_(λ) and the unscattered light *f*_sky_(λ).

In the right-hand side of Equation (3), the first term *f*(λ)*T*(λ) represents the direct attenuation component and the second term *A*(λ)(1 − *T*(λ)) represents the air light component. The former describes the decayed version of *f*(λ) in the atmosphere or the space, while the latter results from scattering by haze and color shifts. The proposed degradation model given in Equation (3) can be considered as a wavelength-extended version of the original hazy image formation model proposed in [1].

Given the UAV image degradation model, the dehazing problem is to restore *f̂*(λ) from *g*(λ) by estimating *T*(λ) and *A*(λ). *f̂*(λ) represents the estimated value of the original haze-free image.

## 3. The Proposed Single UAV Image-Based Dehazing Approach

**Figure 5.**
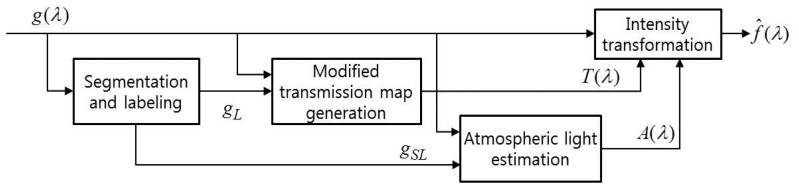
The proposed single UAV image-based dehazing algorithm for enhancing hazy UAV images.

The proposed dehazing algorithm consists of image segmentation and labeling, modified transmission map generation, atmospheric light estimation and intensity transformation modules, as shown in Figure 5.

The label image *g_L_*(λ) is first generated using histogram merging-based classification in the hazy image *g*(λ). Second, the modified transmission map *T*(λ) is generated based on the wavelength-dependent atmospheric turbidity. The corresponding atmospheric light *A*(λ) is then estimated in the labeled version of the sky image *g_SL_*. Finally, the proposed method can significantly enhance the contrast and visibility of a hazy UAV image using the estimated atmospheric light and modified transmission map. As a result, the enhanced image *f̂*(λ) is obtained by adaptively removing hazy components.

Although the wavelength-dependent model was already proposed for underwater image enhancement, the effect of the wavelength may be trivial in the visible spectrum of the air for a UAV image. On the other hand, the proposed work pays attention to the dependency of the wavelength together with the object distance and the amount of scattering. The early work of wavelength-dependent vision through the atmosphere can be found in [26].

### 3.1. Image Segmentation Based on Geometric Classes

The turbidity of the atmosphere varies by the distance of an object and atmospheric degradation factors. Since the conventional transmission map determines the proportion of the light reflected by the object reaching the UAV camera, Tan *et al.* assumed that light traveling a longer distance is more attenuated, yielding the transmission map defined as [16]:

(4) $$T(\lambda )=e^{-\beta d(x,y)}$$

where β represents the scattering coefficient of the atmosphere by color wavelength and *d*(*x, y*) the depth or distance of a point corresponding to the image coordinates (*x, y*).

However, the depth information is difficult to estimate using a single input UAV image. In addition, existing dehazing methods based on the estimation of the atmospheric light and transmission map exhibit various artifacts, such as color distortion, incompletely removed haze and unnaturally enhanced contrast, to name a few [16].

In order to solve these problems, we present a novel transmission map generation method using geometric classes, such as sky, ground and vertical structures. The proposed transmission map provides a theoretical basis to differentiate important regions from the background based on the turbidity and merged classification results.

Existing transmission map generation methods commonly perform image segmentation by minimizing a cost function, such as the ratio cut [27]. Comaniciu *et al.* proposed an image segmentation algorithm using edge information based on the mean shift [28]. Bao *et al.* detected the edge of the input image using a modified canny edge detector with scale multiplication [29]. Erisoglu *et al.* propose a segmentation method to estimate the initial cluster using the *K*-means algorithm [30]. However, most existing methods could not completely solve the problems of noise amplification and edge blurring.

**Figure 6.**
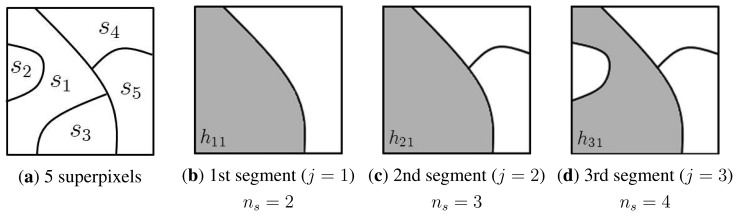
Illustration of the proposed segmentation algorithm: (**a**) five superpixels of an input image and (**b**–**d**) regions containing *s*_1_ for three hypotheses, such as *h_j_*_1_, *j* = 1, 2, 3.

In order to overcome the above-mentioned limitations in existing segmentation methods, the geometric class-based approach in [24] is used for the three-dimensional (3D) context-adaptive processing. The geometric class-based pre-segmentation approach proposed in [24] was inspired by the Hoiem's work in [31], and the proposed approach is an improved version of Yoon's work in [24], where coarse geometric properties are estimated by learning the appearance model of geometric classes. Although Hoiem *et al.* defined a complete set of geometrical classes for segmenting a general scene in their original works, we present a simplified version by selecting only three classes, sky, vertical and ground. Hoiem uses a complete set of geometrical classes, but it causes extremely high computational complexity and an over-segmentation problem. Figure 6 shows a simple illustration to describe the proposed segmentation algorithm.

Figure 6a shows a sample input image decomposed into five superpixels, *s_i_*, *i* = 1,…, 5. In order to assign one of three classes, such as *υ* ∈ {(*S*)*ky*, (*V*)*ertical*, (*G*)*round*}, to each *s_i_*, we perform image segmentation multiple times using different hypotheses. For the first superpixel *s_i_*, Figure 6b–d show regions containing *s*_1_ for the *j*-th hypothesis, such as *h_j_*_1_ for *j* = 1, 2, 3. Let *b_i_* and *b̃_ji_* ∈ {*S*, *V*, *G*} respectively represent labels of *s_i_* and *h_ji_*; then, the most suitable label for *s_i_* maximizes the confidence value, defined as:

(5) $$C(b_{i}=\upsilon |g(\lambda ))=\sum_{j=1}^{3}P(b_{ji}=\upsilon |g(\lambda ),h_{ji})P(h_{ji}|g(\lambda ))$$

where *C* is the label confidence and *P* the likelihood.

For example, the confidence that the first superpixel *s*_1_ is labeled as sky is computed as:

(6) $$\begin{array}{ll} C(b_{1}=S|g(\lambda )) & =P({\tilde{b}}_{11}=S|g(\lambda ),h_{11})P(h_{11}|g(\lambda ))\\ & +P({\tilde{b}}_{21}=S|g(\lambda ),h_{21})P(h_{21}|g(\lambda ))\\ & +P({\tilde{b}}_{31}=S|g(\lambda ),h_{31})P(h_{31}|g(\lambda )) \end{array}$$

**Figure 7.**
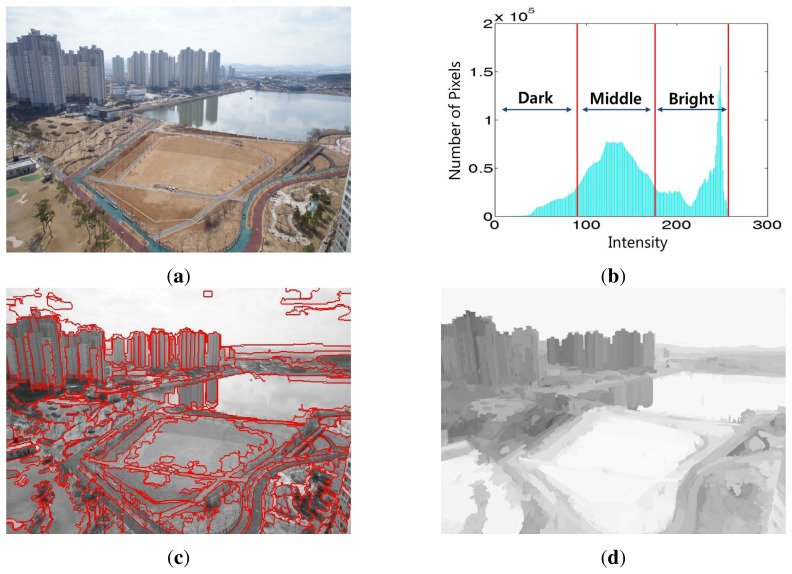
(**a**) A hazy aerial image; (**b**) the corresponding histogram classified into the dark, middle and bright ranges; (**c**) the result of histogram merging-based segmentation; and (**d**) the result of labeling.

The first step of label generation is to estimate superpixels from small, almost-homogeneous regions in the image. Since superpixel segmentation usually preserves object boundaries at the cost of over-segmentation, it can provide accurate boundary information of a subject. We then merge adjacent regions using histogram classification. Next, we uniformly quantize each color channel into 16 levels and then estimate the histogram of each region in the feature space of 16 × 16 × 16 = 4096 bins. The following step merges the regions based on their three classified intensity ranges, such as dark, middle and bright, as shown in Figure 7b.

The similarity measure ρ(*R_A_, R_C_*) between two regions *R_A_* and *R_C_* is defined as:

(7) $$\rho \left(R_{A},R_{C}\right)=\underset{k=1,\ldots K}{\max}\rho \left(R_{A},R_{k}\right)$$

Let *R_C_* and *R_A_* be respectively a region-of-interest and one of its adjacent regions. If there are *K* adjacent regions *R_k_*, for *k* = 1, … , *K, R_C_* is definitely equal to one of the *R_k_*'s. If ρ(*R_A_, R_C_*) is the maximum among *K* similarities ρ(*R_A_, R_k_*), *k* = 1, …, *K*, the two regions are merged as [32]:

(8) $${R^{\prime }}_{C}=R_{C}\cup R_{A}$$

where *R*′*_C_* has the same histogram as that of *R_C_*. This process repeats until there are no more merging regions. The next step computes multiple labels based on simple features, such as color, texture and shape. We generate multiple labels of the hazy image with *n_s_* different regions. We determine the most suitable class for each region using the estimated probability that all superpixels have the same class, which represents each geometric class, such as sky, vertical and ground. Given the hazy UAV image *g*(λ), the superpixel label can be expressed as:

(9) $$C(b_{i}=\upsilon |g(\lambda ))=\sum_{j}^{N_{h}}P(b_{j}=\upsilon |g(\lambda ),h_{ji})P(h_{ji}|g(\lambda ))$$

where *C* represents the label confidence, *b_i_* the superpixel label, *υ* the possible label value, *N_h_* the number of multiple hypotheses and *h_ji_* the region containing the *i*-th superpixel for the *j^th^*^|^ hypothesis. *P*(*h_ji_*|*g*(λ)) represents the homogeneity likelihood and *P*(*b_j_* = *υ*|*g*(λ), *h_ji_*) the label likelihood. Consequently, the sum of the label likelihoods for a particular region and the sum of the homogeneity likelihoods for all regions containing a particular superpixel are normalized.

The proposed segmentation method consists of the following steps: (i) the initial segmentation is performed using superpixels; (ii) the entire histogram is divided into three ranges, as shown in Figure 7b; and (iii) the labeled image is obtained using histogram merging when adjacent segments have similar histograms. In order to simply generate the labeled image, three classes are defined as *υ* ∈ {(*S*)*ky*, (*V*)*ertical*, (*G*)*round*} using color, texture, shape and location and then computing the probability that a new segment has the same class.

Figure 7a shows an input hazy image. Figure 7b shows three classified regions, dark, middle and bright, in the histogram of the input image, and Figure 7c shows the segmentation result using the proposed algorithm. We can obtain the labeled image *g_L_* using the same class of image as shown in Figure 7d.

### 3.2. Spatially-Adaptive Transmission Map

The conventional dark channel prior-based dehazing method generates the transmission map by searching for the lowest intensity in the patch centered at (*x, y*), denoted as Ω(*x, y*), from Equation (3), as [1]:

(10) $$\begin{array}{c} \min\\ c\in \{R,G,B\} \end{array}\left\{\begin{array}{c} \min\\ \left(p,q)\in \Omega (x,y\right) \end{array}\left(\frac{g_{c}(p,q)}{A}\right)\right\}=T(x,y)\begin{array}{c} \min\\ c\in \{R,G,B\} \end{array}\left\{\begin{array}{c} \min\\ \left(p,q)\in \Omega (x,y\right) \end{array}\left(\frac{f_{c}(p,q)}{A}\right)+(1-T(x,y))\right\}$$

where subscript *c* represents one of the RGB color channels and (*p, q*) the pixel coordinates of local patch centers.

Since the minimum intensity in a patch of the ideal haze-free image tends to zero, the transmission map can be computed as:

(11) $$\tilde{T}(x,y)=1-\begin{array}{c} \min\\ c\in \{R,G,B\} \end{array}\left\{\begin{array}{c} \min\\ \left(p,q)\in \Omega (x,y\right) \end{array}\left(\frac{g_{c}(p,q)}{A}\right)\right\}$$

The conventional transmission map results in a halo effect and color distortion in the finally dehazed UAV image, since intensity discontinuity across edges is not considered in the reconstruction process [1]. Although an image matting-based halo effect reduction method has been proposed in the literature, it requires extremely high computational load, which is unsuitable for practical applications. To solve this problem, we generate a modified transmission map by incorporating the classification and labeling results into the conventional transmission map given in Equation (4). The modified transmission map is defined as:

(12) $$T(\lambda )=e^{-\beta g_{G_{i}}\left(\lambda \right)-\alpha }$$

where β represents the scattering coefficient of the atmosphere, λ the wavelength in red (700 μm), green (520 μm) and blue (440 μm) and *α* the wavelength exponent. For the experiment, *α* = 1.5 was used. In Equation (12), since *g_L_*(λ) contains sharp edges, the guided filter is used to generate the context adaptive image *g_G_i__*(λ) that has smoothed edges. The context-adaptive image can be computed by the weighted average of the guided filter as [33]:

(13) $$g_{G_{i}}(\lambda )=\sum_{j}W_{ij}(g_{L})g_{i}(\lambda )$$

where *i, j* are the pixel coordinates, *g*(λ) represents the input hazy UAV image and *g_L_* the guidance label image, and the weighted filter kernel *W_ij_* is given as:

(14) $$W_{ij}(g_{L})=\frac{1}{{\left|\omega \right|}^{2}}\sum_{(i,j)\in \omega_{mn}}\left(1+\frac{\left(g_{L_{i}}(\lambda )-\mu_{mn})(g_{L_{j}}(\lambda )-\mu_{mn}\right)}{\sigma_{mn}^{2}+\varepsilon }\right)$$

where ω*_mn_* is a window centered at (*m, n*), |ω| is the number of pixels in ω*_mn_*, ε is a regularization parameter, μ*_mn_* is the mean and σ*_mn_* is the variance of ω*_mn_*.

The scattering coefficient is determined by the amount of haze, object distance and camera angle. This work uses a camera angle of 60 degrees [34]. The scattering coefficients are defined as:

(15) $$\beta =\{\begin{array}{rr} 0.3324, & \lambda =700\;\mu m(\text{red})\\ 0.3433, & \lambda =520\;\mu m(\text{green})\\ 0.3502, & \lambda =440\;\mu m(\text{blue}) \end{array}$$

In order to generate the transmission map depending on the wavelength, the feature-based labeled image is used. Therefore, the wavelength-dependent transmission map is proposed to adaptively represent hazy images. As a result, the proposed spatially-adaptive transmission map can mitigate the contrast distortion and halo effect problems using the context-adaptive image.

### 3.3. Estimation of Local Atmospheric Light and Intensity Transformation

**Figure 8.**
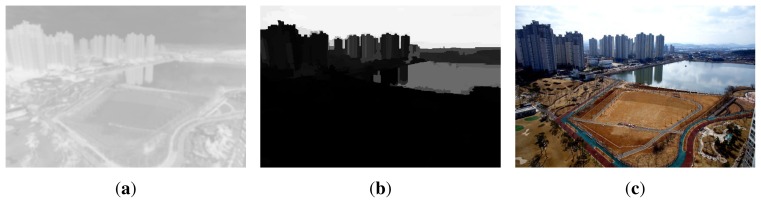
Results of dehazing: (**a**) the transmission map using the proposed method; (**b**) the labeled sky image using the proposed segmentation method; and (**c**) the dehazed image using the proposed modified transmission map and atmospheric light.

Conventional dehazing methods estimate the atmospheric light from the brightest pixel in the dark channel prior of the hazy image. To the best of our knowledge, He *et al.* [1] were the first to raise the issue of color distortion when the atmospheric light is incorrectly estimated from an undesired region, such as a white car or a white building. To address this problem, we estimate the atmospheric light using the labeled sky image that can be generated from the ‘sky’ class, as shown in Figure 8b. As a result, the atmospheric light can be estimated as:

(16) $$A(\lambda )=\max(g(\lambda )\cap g_{SL})$$

where *g*(λ) represents the hazy image and *g_SL_* the labeled sky image. This procedure can mitigate the color distortion problem in conventional dehazing methods.

Given the adaptive global atmospheric light *A*(λ) and the modified transmission map *T*(λ), the dehazed image is finally restored as:

(17) $$\hat{f}(\lambda )=\frac{g(\lambda )-A(\lambda )}{T(\lambda )}+A(\lambda )$$

Figure 8a shows the proposed transmission map of Figure 7a. As a result, the proposed spatially-adaptive transmission map has continuous intensity values in the neighborhood of boundaries. Figure 8b shows the labeled sky image using the proposed segmentation method. Figure 8c shows the dehazed image using the modified transmission map in Figure 8a. As shown in Figure 8c, the proposed method significantly enhanced the contrast of the hazy image without color distortion or unnatural contrast amplification.

## 4. Experimental Results

In this section, we show the experimental results to compare the performance of the proposed dehazing algorithm with conventional methods.

**Figure 9.**
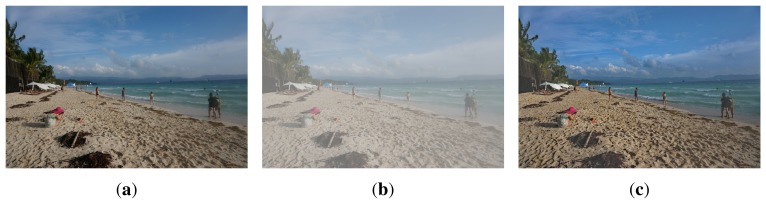
Performance evaluation of color restoration using a simulated hazy image: (**a**) original haze-free image; (**b**) the simulated hazy image; and (**c**) the dehazed image using the proposed method.

In order to demonstrate the performance of original color restoration, a set of hazy images are first generated by simulation. The test images are then enhanced using the proposed dehazing algorithm to evaluate the accuracy of color restoration, as shown in Figure 9.

**Figure 10.**
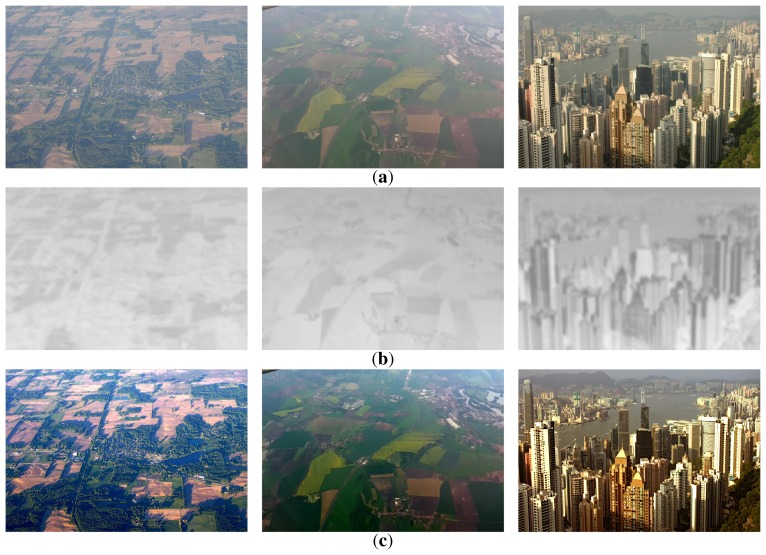
Experimental results of the proposed dehazing method: (**a**) input hazy UAV images; (**b**) the modified transmission maps; and (**c**) the dehazed UAV images using the proposed method.

Figure 10a shows three hazy unmanned aerial vehicle (UAV) images; Figure 10b shows the modified transmission map using the proposed method; and Figure 10c shows the dehazed results using the proposed method. As shown in Figure 10c, the proposed dehazing method preserves fine details without color distortion. If there are no sky regions in an image, as shown in Figure 10, the atmospheric light can be simply estimated using the maximum intensity value.

**Figure 11.**
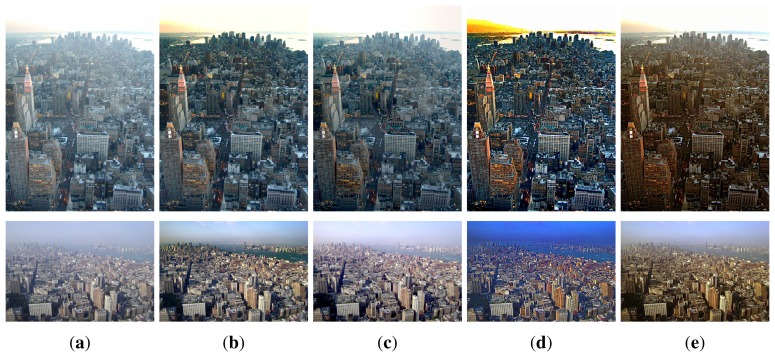
Experimental results of various dehazing methods: (**a**) input hazy images; (**b**) dehazed images using He's method; (**c**) dehazed images using Fattal's method; (**d**) dehazed images using Tan's method; and (**e**) dehazed images using the proposed method.

The performance of the proposed dehazing algorithm is compared with three existing dehazing methods. Figure 11a shows two hazy images, and Figure 11b shows the dehazed images using the conventional dark channel prior-based method [1], where the hazy components were removed at the cost of color distortion in the sky region. Figure 11c shows dehazed images using Fattal's method proposed in [15], where the haze is not completely removed in regions far from the camera. Figure 11d shows dehazed results using Tan's method proposed in [16], where color distortion is visible. As shown in Figure 10e, the dehazed image using the proposed method shows significantly improved image quality without color distortion or unnaturally amplified contrast. Furthermore, the proposed method maintains the haze in the sky and removes haze surrounding the buildings.

In order to justify the performance of dehazing, Tarel *et al.* compared a number of enhancement algorithms [35]. On the other hand, the proposed work is evaluated in the sense of both subjective and objective manners using visual comparison in Figure 11 and quantitative evaluation in Table 2, respectively. We compared the performance of the proposed dehazing algorithm with three existing state-of-the-art methods in the sense of the visibility metric proposed by Zhengguo. The visibility metric is used to calculate the contrast-to-noise ratio (CNR) of dehazed images.

**Figure 12.**
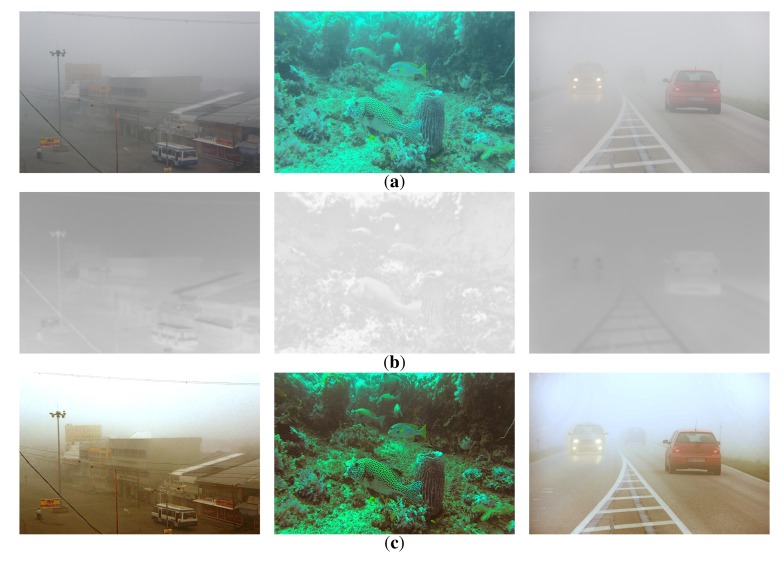
Experimental results of the proposed dehazing method: (**a**) input hazy images acquired by a video surveillance camera, an underwater camera and a vehicle black box camera, respectively; (**b**) the modified transmission maps; and (**c**) the dehazed images using the proposed method.

Figure 12a shows hazy images acquired by a video surveillance camera, an underwater camera and a vehicle black box camera. Figure 12b shows the modified transmission maps using the proposed method, and the results of the proposed dehazing method are shown in Figure 12c. Based on the experimental results, the proposed dehazing method can successfully restore the original color of the scene with a moderately hazy atmosphere, but its dehazing performance is limited with a severe amount of haze in the atmosphere.

**Figure 13.**
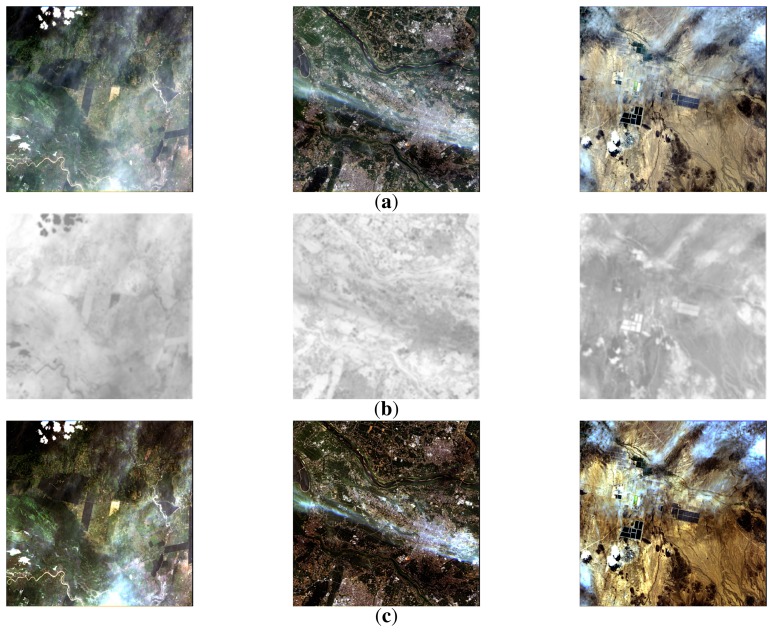
Experimental results of the proposed dehazing method: (**a**) input hazy satellite images courtesy of the Korea Aerospace Research Institute (KARI); (**b**) the modified transmission maps; and (**c**) the dehazed images using the proposed method.

Figure 13a shows three satellite images acquired by the Korea Aerospace Research Institute (KARI). Figure 13b shows the modified transmission maps, and Figure 13c shows the dehazed results using the proposed method. Based on the experimental results, the proposed dehazing method can successfully restore the original color of the scene with a moderately hazy atmosphere, except with an excessive amount of haze, such as thick cloud.

## 5. Conclusions

In this paper, we presented a spatially-adaptive dehazing algorithm based on a wavelength-adaptive hazy image formation model. As a major contribution, the proposed wavelength-based dehazing method can mitigate the color distortion problem in conventional methods. By incorporating the wavelength characteristics of light sources into the UAV image degradation model, the proposed transmission map removes hazy components in the input image. Another contribution is that the proposed algorithm needs neither additional optical equipment nor *a priori* distance estimation.

Experimental results show that the proposed dehazing algorithm can successfully restore the original color of the scene containing the wavelength-dependent scattering of atmosphere. This proposed algorithm can be used for various applications, such as video surveillance systems, intelligent driver assistant systems and remote sensing systems. The proposed wavelength-adaptive dehazing algorithm is particularly suitable for preprocessing multispectral registration of satellite images for enhancing aerial images with various types of haze, fog and cloud.

## Figures and Tables

**Table 1.** Mean RGB values of the white region in Figures 1 and the corresponding color alignment measure (CAM) values.

**Image Type**	*C_r_*	*C_g_*	*C_b_*	**CAM**
Figure 1a	0.9044	0.9049	0.8884	0.0044
Figure 1b	0.7562	0.7640	0.7648	0.0024
Figure 1c	0.7089	0.7147	0.7199	0.0021
Figure 1d	0.6657	0.6711	0.6743	0.0019

**Table 2.** Quantitative performance of the proposed and three state-of-the-art dehazing methods in the sense of both contrast-to-noise ratio (CNR) and the measure of enhancement (ME).

**Input Image**	**Dehazing Method**	**CNR**	**ME**
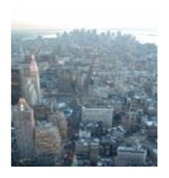	Haze Image	53.0588	-
	He's Method [1]	68.8744	15.8156
	Fattal's Method [14]	71.8029	18.7441
	Tan's Method [15]	84.9931	31.9343
	Proposed Method	72.4338	19.3750

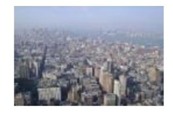	Haze Image	58.8830	-
	He's Method [1]	72.8756	13.9626
	Fattal's Method [14]	67.5625	8.6795
	Tan's Method [15]	88.5869	29.7039
	Proposed Method	81.4254	22.5424
